# Intra-articular hyaluronic acid for treatment of osteoarthritis: a nationwide study among the older population of Taiwan

**DOI:** 10.1186/1472-6963-8-24

**Published:** 2008-01-28

**Authors:** Hsiu-Yun Lai, Yu-Chun Chen, Tzeng-Ji Chen, Li-Fang Chou, Liang-Kung Chen, Shinn-Jang Hwang

**Affiliations:** 1 Department of Family Medicine, Taipei Veterans General Hospital, Taipei, Taiwan; 2 Center for Geriatrics and Gerontology, Taipei Veterans General Hospital, Taipei, Taiwan; 3 Department of Family Medicine, School of Medicine, National Yang-Ming University, Taipei, Taiwan; 4 Institute of Biomedical Informatics, School of Medicine, National Yang-Ming University, Taipei, Taiwan; 5 Department of Public Finance, National Chengchi University, Taipei, Taiwan

## Abstract

**Background:**

Although intra-articular treatment with hyaluronic acid (HA) for symptomatic osteoarthritis has become widely accepted in recent decades, the pattern of its use has seldom been reported. We have explored the epidemiology of intra-articular HA treatment in Taiwan by using the rich data source from nationwide insurance claims.

**Methods:**

Taiwan's National Health Insurance (NHI), which covers 97% of inhabitants, offers extensive hospitalisation and ambulatory care. We identified the beneficiaries aged 60 years and older who received intra-articular HA within the NHI during 2004. The number of visits in which HA was administered were analysed by patient's age and gender and by the physician's specialty and practice site.

**Results:**

Among the 73,410,777 ambulatory visits by 2,909,219 beneficiaries aged 60 years and older in 2004, 35,782 (1.2%) patients received intra-articular HA treatment in 205,012 (0.3%) visits. The highest prevalence of HA use was in the 70–79 year age group in both sexes. Women received intra-articular HA treatment more frequently than men in all age groups, especially in the 60–69 and 70–79 year groups (1.6% vs. 0.5%, 2.2% vs. 1.0%, respectively). Most intra-articular HA procedures were performed by orthopaedic surgeons (75.1%) and physical medicine and rehabilitation physicians (15.2%), and at metropolitan hospitals (34.5%) and local community hospitals (38.2%).

**Conclusion:**

One out of 100 older patients in Taiwan received intra-articular HA treatment for osteoarthritis of the knee during the course of the year. There were age-gender differences in use of HA treatment. The completion rate of this treatment in our study was high, and thus intra-articular HA might be a good alternative for patients for whom conventional treatment fails. Further research is needed to examine the age-gender differences in use of intra-articular HA in Taiwan.

## Background

Osteoarthritis of the knee is one of the leading causes of disability among the older population. Beside functional activities, it affects social relationships, body image and emotional well-being [1,2]. In Western countries, 7–18% of older people have symptomatic osteoarthritis of the knee [3-5]. A population-based study in China also found that symptomatic osteoarthritis of the knee occurs in 15% of women and 6% of men aged 60 and older [6]. The pain arising from osteoarthritis can be treated with several kinds of drugs: paracetamol, non-steroidal anti-inflammatory drugs (NSAIDs), opioid analgesics, glucosamine salt, chondroitin sulphate, diacerein, corticosteroids, hyaluronic acid (HA) and capsaicin [7]. Most of these drugs are for either oral or topical use; only corticosteroids and HA can be administered intra-articularly. Because of its viscoelasticity, HA may replace synovial fluid and protect the cartilage [8,9]. Intra-articular HA treatment for osteoarthritis of the knee was approved by the US Food and Drug Administration in 1997. In comparison with NSAID and corticosteroid, the better tolerability and fewer adverse effects of HA have made it widely accepted in recent decades [8,10,11].

Most studies of intra-articular HA treatment examine its effectiveness, but few consider the pattern of its use. In Taiwan, HA treatment is reimbursed within the National Health Insurance (NHI) system. Because complete NHI claims are available in electronic form to researchers, we could investigate the nationwide age- and sex-specific prevalences and patterns of use of intra-articular HA in Taiwan. Such information might elucidate the help-seeking behaviour of patients and the preferences of physicians in treating osteoarthritis of the knee, and could help to allocate medical resources in health policy-making by adjusting the reimbursement of this procedure in the NHI.

## Methods

### Data sources

The NHI program in Taiwan started in 1995 and covered 22,134,270 beneficiaries (about 97% of inhabitants) at the end of 2004 [12]. Since 1999, the Bureau of National Health Insurance has released the claims data to the National Health Research Institutes (NHRI) for research use under the project of National Health Insurance Research Database [13]. The electronic datasets contain visit and prescription details, including the encrypted identification numbers of beneficiaries and healthcare facilities. With the approval of NHIRD for the research use of anonymised datasets, the researchers must also sign a user agreement of obeying the regulations of NHIRD and acknowledging the NHIRD in their publications.

Since 1999, the NHI has reimbursed the mode of intra-articular HA treatment for any patient whose osteoarthritis of the knee is poorly responsive to conventional analgesics but who is not yet eligible for total knee arthroplasty. Physicians can decide by clinical judgement whether patients meet the criteria for intra-articular HA treatment. During the course of such treatment, the patient cannot concomitantly receive NSAIDs, intra-articular corticosteroids or rehabilitation by physical modalities. Only two courses are reimbursable for each patient in a year. Each course includes 3–5 sessions of injection, depending on the molecular weight of the HA product. We obtained the dataset from the NHIRD containing the complete claims of beneficiaries aged 60 and older in 2004.

Ten intra-articular HA products were available within the NHI in 2004. Because physicians had to prescribe HA products to the patients before each injection, we could identify the patients directly from the prescription records. Those who received no injection at that visit would have no HA record.

### Analyses

The annual age-sex prevalence of patients receiving intra-articular HA treatment was analysed in three age groups: 60–69, 70–79, and 80 years and older. We further calculated the total number of intra-articular HA visits per patient during the year. The data were also stratified by the specialties of physicians and the accreditation level of the healthcare facilities. There were 24 specialties and 22 subspecialties. We arbitrarily grouped the practitioners with no specialist title together with family physicians into 'general practices'. A healthcare facility enters into a contract with the NHI in one of 4 categories: academic medical centre, metropolitan hospital, local community hospital, or physician clinic.

The data were imported into Microsoft SQL Server 2005 (Microsoft Corp., Redmond, WA, USA) for computation. The denominator was the number of patients aged 60 years and older who had been insured within the NHI in 2004. Descriptive statistics including means, standard deviations and percentages were calculated.

## Results

At the end of 2004, a total of 2,909,219 beneficiaries aged 60 years and older were insured within the NHI in Taiwan: 1,459,513 (50.2%) women, 1,449,688 (49.8%) men and 22 persons of unknown sex. The average age was 70.9 ± 6.6 years: 72.8 ± 6.8 years for men and 70.2 ± 6.4 years for women. From 73,410,777 ambulatory visits by the older population in 2004, we identified 35,782 (1.2%) patients receiving intra-articular HA treatment in 205,012 (0.3%) visits.

Stratified by age, the highest prevalence of HA use was in the 70–79 group (1.5%) and the lowest was in the 80 years and older group (1.0%) (Table 1). Women were more likely to receive intra-articular HA treatment than men (1.8% vs. 0.7%), especially in the 60–69 (1.6% vs. 0.5%) and 70–79 (2.2% vs. 1.0%) year groups. The gender difference diminished with age.

**Table 1:** Age-sex prevalence and utilization of patients receiving intra-articular hyaluronic acid treatment in 2004

	No. of NHI beneficiaries			No. of HA patients (%)		HA visits per patient in 2004, mean ± SD	
Age	Male	Female	Total	Male	Female	Male	Female

60–69	710,174	767,070	1,477,244	3,421 (0.5)	12,587 (1.6)	5.6 ± 3.3	5.7 ± 3.1
70–79	547,817	492,487	1,040,664	5,298 (1.0)	10,715 (2.2)	5.7 ± 3.5	5.8 ± 3.4
≧ 80	191,697	199,614	391,311	1,654 (0.9)	2,192 (1.1)	5.6 ± 3.5	5.4 ± 3.2

**Figure 1:**
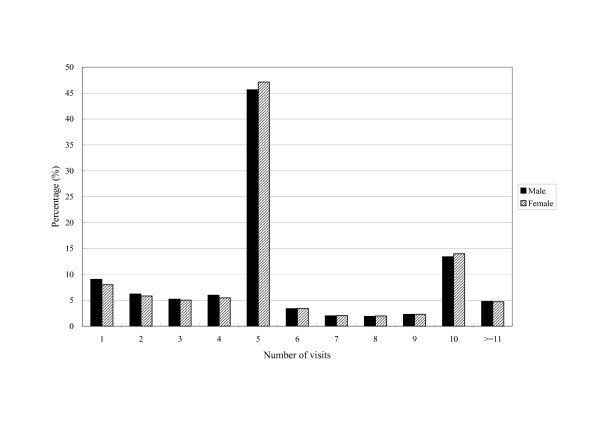
Sex-specific frequency distribution of visits for intra-articular hyaluronic acid treatment per patient in 2004 (a total of 205,012 visits by 35,782 patients).

**Figure 2:**
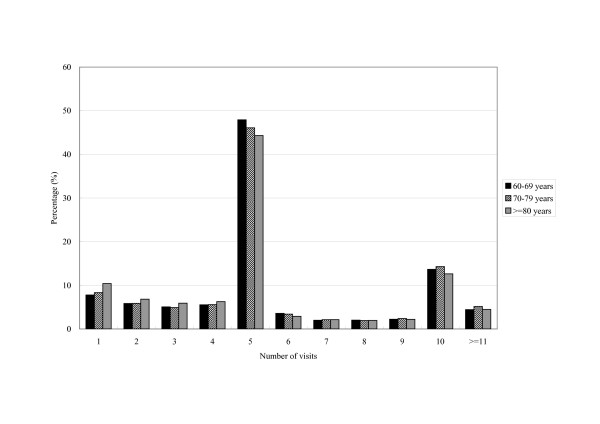
Age-specific frequency distribution of visits for intra-articular hyaluronic acid treatment per patient in 2004 (a total of 205,012 visits by 35,782 patients).

On average, a patient had 5.7 ± 3.3 visits for intra-articular HA treatment during the year. Almost half the patients received the treatment 5 times in the year and 13.8% received it 10 times. The frequency distribution showed no marked sex and age differences (Figure 1 and 2).

Most of the intra-articular HA treatment occurred in orthopaedic (75.1%) and rehabilitation (15.2%) clinics, and only 1.8% in general practices (Table 2). In all ambulatory visits of beneficiaries 60 years and older in 2004, academic medical centres accounts for 14.8 % visits, metropolitan hospitals for 16.4%, local community hospitals for 17.5% and physicians clinics for 51.3%. However, nearly three-quarters of intra-articular HA treatment were performed in metropolitan hospitals (34.5%) and local community hospitals (38.2%) (Table 3).

**Table 2:** Distribution of visits for intra-articular hyaluronic acid treatment in 2004 by specialty

	No. of visits (%, n = 205,012)	No. of patients* (%, n = 35,872)
Orthopaedics	153,980 (75.1)	27,292 (76.1)
Rehabilitation	31,053 (15.2)	5,323 (14.8)
Rheumatology	7,268 (3.6)	1,399 (3.9)
General surgery	4,180 (2.0)	1,072 (3.0)
General practice	3,666 (1.8)	894 (2.5)
Internal medicine	2,487 (1.2)	621 (1.7)
Neurosurgery	1,067 (0.5)	236 (0.7)
Pain management	952 (0.5)	207 (0.6)
Others	359 (0.2)	96 (0.3)

*Because a patient might visit several specialties, the total of all percentages in this column exceeded 100%.

**Table 3:** Distribution of visits for intra-articular hyaluronic acid treatment in 2004 by contracted category of healthcare facility

Accreditation level	Overall No. of visits (%, n = 72,665,294)	No. of HA visits (%, n = 205,012)	No. of HA patients* (%, n = 35,872)
Academic medical centres	10,736,652 (14.8)	33,074 (16.1)	6,425 (17.9)
Metropolitan hospitals	11,932,861 (16.4)	70,625 (34.5)	11,760 (32.8)
Local community hospitals	12,730,923 (17.5)	78,268 (38.2)	14,548 (40.6)
Physician clinics	37,264,858 (51.3)	23,045 (11.2)	4,356 (12.1)

*Because a patient might visit several healthcare facilities, the total of all percentages in this column exceeded 100%.

## Discussion

Our nationwide survey offered a very rich source of data concerning intra-articular HA treatment for osteoarthritis of the knee among the total older population of Taiwan. Although the prevalence of osteoarthritis of the knee generally increases with age [14], our study revealed that the utilization of intra-articular HA treatment in Taiwan did not fully correlate with the patient's age. People aged 70–79 years tended to receive HA treatment most frequently, and people aged 80 years and older least. This age-specific pattern of HA use is similar to that of total knee arthroplasty among osteoarthritic patients. A previous study showed that the probability of undergoing total joint arthroplasty increased from 62 to 81 years but fell after 82 years [15]. Another study found that most of those aged 75 years and older who might benefit from knee arthroplasty had not been referred to rheumatological or orthopaedic services [16]. Because the oldest people generally have multiple comorbidities, primary care physicians might hesitate about referral. As HA treatment has a low incidence of severe adverse events and good tolerability compared with NSAIDs and corticosteroids [8,10,11], it might be a relatively safe alternative for the oldest patients.

The prevalence, incidence, symptoms and severity of osteoarthritis differ not only with age but also between sexes [17]. Several studies have shown that women are more likely than men to have both radiographic and symptomatic osteoarthritis of the knee. The female-to-male ratio for radiographic osteoarthritis of the knee varies from 1.5 to 4.0 [2,14], and that of symptomatic osteoarthritis of the knee from 1.6 to 1.7 [5,18,19]. In addition, a community study has shown that the sex difference is more obvious in the Chinese population [6]. In our study, such a sex difference existed in the use of intra-articular HA treatment. This might be due to the difference in disease prevalence and severity between the sexes. A difference between sexes in willingness to receive treatment might also play a role. One study showed that women underwent total knee arthroplasty at a more advanced stage of their disease [20]. Even with equal willingness to undergo surgery, fewer women than men had discussed the possibility of arthroplasty with a physician though the degree of underuse was more than three times as great in women as in men [18]. These findings might suggest that women are more inclined to non-operative treatment or that physicians are more likely to offer intra-articular HA treatment to women.

A previous study also showed that the sex difference in the use of arthroplasty of knee varied with age. Tennant and colleagues reported that women aged over 55 years made more demands for knee arthroplasty than men of the same age and the discrepancy increased with age [16]. In our study, we found that the sex difference in the use of intra-articular HA treatment diminished with age. A possible reason might be the increasing use of arthroplasty among aged women. The interaction between age and sex could only be confirmed in longitudinal studies spanning longer periods. Survey-based studies of patient preferences may also be needed to help understand whether patient treatment preferences can explain the gender/age findings in this study.

In 2004, most of the HA products available within the NHI in Taiwan were of lower molecular weight. A course of five injections was suggested to achieve a better clinical effect. Many patients in our study received intra-articular HA treatment 5 or 10 times in one year. We might presume that most patients completed the treatment course(s), showing good tolerance and adherence to intra-articular HA. Although its efficacy is currently controversial within academia, HA is generally thought to be more tolerable than operative procedures and less invasive than NSAIDs [8,10,11].

In our study, nearly three-quarters of intra-articular HA treatment occurred in metropolitan hospitals and local community hospitals, what was disproportionate to the all visits. The results may reflect either patients' or physicians' preference. Further research may help provide detailed information.

Our nationwide claims-based study had some limitations. The one-year cross-sectional survey could not provide information about trends in intra-articular HA use. The claims contained no data about residence, socio-economic background, severity of the disease, functional status or the response to treatment. Furthermore, because the diagnostic coding by ICD-9-CM (the International Classification of Diseases, Ninth Revision, Clinical Modification) in the ambulatory claims was not always precise, we could not identify all patients with osteoarthritis of the knee to calculate the prevalence of this condition or the percentage of patients with HA injections among all patients suffering from it.

## Conclusion

The high usage and completion rates of intra-articular HA treatment reflected high tolerance of the treatment in geriatric ambulatory care. Further research is needed to elucidate the effectiveness and adverse effects of this important osteoarthritic knee treatment in the elderly population.

## List of abbreviations

HA: Hyaluronic acid

NHI: National Health Insurance

NHIRD: National Health Insurance Research Database

NSAID: Non-steroid anti-inflammatory drugs

## Competing interests

The author(s) declare that they have no competing interests.

## Authors' contributions

HYL conceived and carried out the study, performed the statistical analysis and drafted the manuscript. YCC participated in the design of the study and helped to perform the statistical analysis. LFC and LKC participated in the design of the study and helped to interpret findings. TJC and SJH participated in the design and coordination of the study and helped to draft the manuscript. All authors read and approved the final manuscript.

## Pre-publication history

The pre-publication history for this paper can be accessed here:


